# Occupational injuries and fatalities in a tanzanite mine: Need to improve workers safety in Tanzania

**DOI:** 10.11604/pamj.2013.16.120.3420

**Published:** 2013-11-27

**Authors:** Respicious Boniface, Lawrence Museru, Victoria Munthali, Ronald Lett

**Affiliations:** 1 Muhimbili Orthopaedic Institute (MOI), Dar es Salaam, Tanzania; 2 Injury Control Centre Tanzania (ICCT); 3 Muhimbili University of Health and Allied Sciences (MUHAS), Dar es Salaam, Tanzania; 4 Canadian Network for International Surgery, Vancouver, Canada; 5 Surgery Department, McGill University of British Columbia

**Keywords:** Injuries, mine workers, Mererani, Tanzania

## Abstract

**Introduction:**

Work related injuries are common, and the mining industry accounts for a significant proportion of these injuries. Tanzania is among the countries with high rates of mining injuries, nevertheless pre-hospital care is almost non existant and health care service deliveries are poor. This study sought to identify factors associated with injuries and fatalities among miners in Mererani, Tanzania.

**Methods:**

A Cross - Sectional study of miners who sustained injuries and seen at Mererani health centre between January 2009 and May 2012.

**Results:**

In the selected period 248 injury patients were seen. All were males, and 54% were between 18 - 30 years age-group. Almost all (98.7%) didn’t use protective gears at work, and worked for more than 12 hours daily. Falling rocks were the leading cause of injury (18.2%), and majority sustained multiple injuries (33%). Of the patients seen, 41.3% died. The following were more likely to die than others; Primary education (p = 0.04), Less than 5 years work experience (p = 0.000), unintentional injuries (p = 0.000), fall injuries (p = 0.000) and sustaining multiple injuries (p = 0.000).

**Conclusion:**

The burden of injuries and fatalities demonstrated in this study, point to the need for implementation and monitoring of the use of safety equipment and operating procedures of the mines by government and other regulatory authorities. Initiation of pre hospital care at the mines and improved emergency medical service delivery at health centers in Tanzania.

## Introduction

According to the International Labour Office statistics, 120 million occupational accidents occur annually at workplaces worldwide [1], of these, 210,000 are fatal accidents. Occupational injuries take a considerable socioeconomic toll of workers, companies and society, whereas they draw little public attention. Mining industry accounts for a significant proportion of these injuries, particularly fatal injuries and mining has been considered one of the world's most dangerous occupations [2].

A study based on fatality information during the 16-years period from 1980 to 1995 in the United States, reported that, mining had the highest fatal occupational injury rate [3]. Michelo et al (2009) reported 165 injuries and 20 fatalities from January 2005 to May 2007 in one of the largest copper mining in Zambia [4]. The incidence of injury remains high although some improvements in work conditions have been made as a result of catastrophic events and scientific progress [5]. Higher rates of injuries and fatalities have been reported for low-income countries in Africa and Asia compared to Europe and America [6], in Sub-Saharan Africa for example, more than 54000 fatal occupational accidents happen annually compared to 16000 fatal occupational accidents in Europe and America.

Small-scale mining in Tanzania is to a large extent unregulated, and therefore not safe. For example in Mererani, in 2002, some 48 miners were suffocated to death when a compressor used to pump in clean air failed to work. In 2006 a miner was killed by falling loose rocks, and in March 2007 the death of three miners in the same area was attributed to collapsed pits. Also at Mererani in 2008 at least 65 miners drowned to death after floods swept through pits and tunnels [7]. And these are but just a few of deaths caused by mining accidents in the country.

Common causes of fatal injury include rock fall, fires, explosions, mobile equipment accidents, and falls from height, entrapment, suffocation and flooding of underground workings [8]. Several studies have revealed the role of human behavior in mine injuries. Shaw et al (1989) identified human error to be the major causative factor in mine injuries [9]. They concluded that while a few injuries are caused by a single factor, human error was the most significant contributing factor and accounted for 93% of the total injuries.

Individual factors which concern many employed people such as age, safety performance, working condition, safety environment, management and supervision, emotional stability, job involvement, job satisfaction and job stress have been identified as risk factors for occupational injuries in previous studies [10].

As a result of the fatalities and injuries occurring in mines every year, the world is paying heavily in terms of both human suffering and economic losses. In order to develop effective preventive measures, information on associated factors is required. The purpose of this study was to investigate factors associated with injuries and fatalities among miners working in the Mererani Tanzanite Mines in, Tanzania.

## Methods

**Study Setting**. This study was conducted at Mererani heath centre, a government health facility in Mererani Township, Manyara region in Northern Tanzania. The heath centre has 22 bed capacities, six clinical officers and 15 nurses who provide care of patients daily. Mererani is home to about 55,000 people, the majority of whom work in mines in the Mererani Tanzanite hills four kilometers from the mining settlement of Mererani Township. There are about 500 working small mines in Mererani, employing about 40000 miners in the mining process of Tanzanite.

**Study Design**. This was a cross-sectional study of miners who sustained injury and seen at Mererani health centre between January 2009 and May 2012.

**Data Collection and Management**. Trained research assistants used a structured questionnaire to collect information from the study participants in a face-to-face interview, and from health facilities medical records. Information were collected on socio-demographic factors, details such as employment date, date of injury, time of injury, etiology, place of injury, nature of injury, and victim's outcome. Data were double-entered into Epi info version 3.5.1 (CDC, Atlanta, USA). Statistical analysis was conducted using STATA version 11 (Statacorp, College Station, USA).

**Statistical Analysis**. All variables were categorized and described using frequency distribution. The dependent variable mortality was categorized as a dichotomous variable (death or no-death). Bivariate associations were described using chi-square tests. A variable with (p=0.05) with mortality was considered to be statistically significant.

**Ethical Consideration**. Ethical approval for the study was obtained from the Muhimbili University of Health and Allied Sciences Research Ethics Committee

## Results

**Figure 1:**
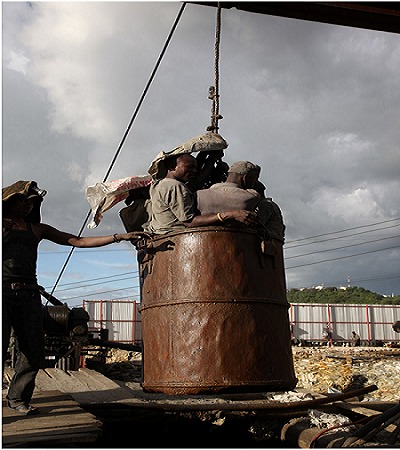
Tanzanite miners at Mererani in a barrel, with no protective gears

In the selected period a total of 248 Mererani mine injury patients were seen. All were males, and 73% were residence of Kilimanjaro region. Majority were between 18 years and 30 years age-group (54%). More than half (52.1%) were married, and 62.1% had been to primary school (Table 1). Majority (95.9%) perceived the work condition as being fair. Almost all (98.7%), didn’t use protective gears at work (Figure 1), and that they work for more than 12 hours daily, 73.4% had work experience of less than 5 years. Of the 248 patients seen, 8.1% were admitted, 19.4% treated and discharged, 31.2% referred to higher level and 41.3% died during treatment or dead on arrival.

**Table 1:** Demographic characteristics of study participants

Characteristic	N	(%)
**Age**		
18 to 30 years	118	54.1
31 to 50 years	91	41.7
>50 years	9	4.1
Total	218	100.0
**Marital status**		
Divorced	5	2.3
Married	114	52.1
Never married	100	45.7
Total	219	100.0
**Education level**		
No education	27	13.3
Primary	126	62.1
Secondary	50	24.6
Total	203	100.0
**Cigarette smoker**		
No	84	40.2
Yes	125	59.8
Total	209	100.0
**Alcohol drinker**		
No	138	63.4
Yes	78	36.6
Total	213	100.0
**Work safety condition**		
Bad	4	1.6
Fair	238	95.9
Good	6	2.4
Total	248	100.0
**Work experience**		
1 to 5 years	160	73.4
> 5 years	58	26.6
Total	218	100.0

N = Number

### Causes of injury

The top five causes of injuries were; falling rocks (18.2%), explosions (16.9%), falls (16.1%), suffocation (13.2%), and assault (13.2%). Majority sustained multiple injuries 33.3%, cut/open wounds 19.1%, and head/neck injuries 17.9% (Table 2).

**Table 2:** Factors associated with mortality

Characteristic	Death N (%)	No death N (%)	P value
**Age**			0.165
18 to 30 years	52 (59.8)	66 (50.4)	
31 to 50 years	30 (34.5)	61 (46.6)	
>50 years	5 (5.8)	4 (3.1)	
**Education level**			0.04**
No education	5 (6.5)	22 (17.6)	
Primary	51 (66.2)	74 (59.2)	
Secondary	21 (27.3)	29 (23.2)	
**Work safety condition**			0.856
Bad	2 (1.9)	2 (1.4)	
Fair	97 (95.1)	140 (96.6)	
Good	3 (2.9)	3 (2.1)	
**Work experience**			0.000**
< 5 years	69 (73.4)	76 (61.3)	
≥ 5 years	25 (26.6)	48 (38.7)	
**Intent**			0.000**
Undetermined	14 (13.7)	2 (1.4)	
Unintentional	87 (85.3)	116 (80.0)	
Intentional	1 (0.9)	27 (18.6)	
**Cause of injury**			0.000**
Assault	2 (2.0)	30 (21.1)	
Collapse	5 (5.0)	8 (5.6)	
Crushed	0 (0.0)	5 (3.5)	
Drowning	7 (7.0)	0 (0.0)	
Explosion	9 (9.0)	32 (22.5)	
Falling equipments	12 (12.0)	1 (0.7)	
Suffocation	5 (5.0)	27 (19.0)	
Falling rocks	10 (10.0)	34 (23.9)	
Falls	37 (37.0)	2 (1.4)	
Unknown	13 (13.0)	3 (2.1)	
**Nature of injury**			0.000**
Burn	4 (6.8)	11 (8.5)	
Chest injury	6 (10.2)	7 (5.4)	
Cut/Open wound	3 (5.1)	43 (25.4)	
Drowning	7 (11.9)	0 (0.0)	
Fracture	8 (13.6)	2 (1.5)	
Multiple injuries	23 (38.9)	40 (30.8)	
Head/Neck injury	8 (13.6)	26 (20.0)	

* P value ≤0.05; N = number

### Factors associated with mortality

The following were more likely to die than others (Table 2); Primary education level (p = 0.04), less than 5 years work experience (p = 0.000), unintentional injuries (p = 0.000), fall injuries (p = 0.000) and sustaining multiple injuries (p = 0.000).

## Discussion

The number of injuries and fatalities reported in this study has important implications for informing policy makers aiming at reducing injuries and fatalities occurring in the mines.

Majority of the injured patients in this study were between 18 - 30 years age-group (54%), similar to what has been reported in other studies [11, 12]. This is young and most active age-group which contributes to the risk taking behavior, and most of the miners working in Mererani belong to this age-group. Thus these results suggest that supervisors should aim to increase awareness among all workers, particularly those aged 18 - 30 years who take risks at work. Other studies however have reported higher risk of injuries among older workers [13, 14], attributing their injuries to decrease in physical and mental abilities which may in turn alter the ability to notice work environment hazards.

All injured patients in this study admitted to work for more than 12 hours daily, and they did not think that there situation was dangerous. Prolonged continuous working periods decrease workers attention and concentration levels resulting in increased risk of injuries. This is similar to what was reported in a study done in USA, in which it was revealed that working for more than 8 hours per shift resulted in a 61% increased risk for occupational injuries [15]. Almost all patients (98.7%) didn’t use protective gears at work. Personal protective gears put a barrier between the worker and the hazard thereby protecting the worker from injury. Mining work is risky and for a worker to work without protective gear is taking unnecessary risk. These findings are similar to what was reported in Zimbabwe [16], in which it was found that inadequate personal protective equipment and clothing were significantly associated with getting severely injured at the mine.

Similar to what was reported in Zambia copper mines [4], falling rocks was the most common cause of injuries in this study accounting for 18.2%. Other top causes were: explosion (16.9%), falls (16.1%), assault and suffocation each accounting for (13.2%). These incidents may be due to the use of unsafe equipments like using barrel mounted on a cable (Figure 1) for rising and lowering miners in a shaft, lack of safety training, carelessness and dangerous procedures. Majority of ongoing mining operations in Mererani use rudimentary tools and lack enforcement of safety standards.

The overall mortality rate among injured patients in this study was 41.3%, which is very high. Education level was not a protective factor for mortality in this study, as those with primary school education were more likely to die than those with no formal education. This can be due to the fact that majority of study participants (62.1%) had primary school education. Other investigators [17] have reported high risk of mortality among workers with no formal education, these are more likely to commit human errors as they are less well prepared to undertake job training and follow safe working procedures.

Results from this study show that majorities (73.4%) had a work experience of less than 5 years, and the risk of mortality was more among those with less than 5 years experience. This can be due to the fact that, those with lower job experience have less knowledge of workplace hazards, and they are exposed to more hazardous working conditions than those with higher job experience [18]. Majority of high experienced miners in Mererani are supervisors and so are less likely to be exposed to high risk working condition.

The most frequent cause of fatal injuries was falls. This includes falling out of the barrel that were being raised or lowered in a shaft, slipping and falling, or being knocked off a piece of equipment and falling. Poor technology used in extraction and recovery of minerals and the failure to invest in safe working equipment and tools threaten miners lives in the country. The overriding issue is that the mines are unsafe and that workers are not provided with safety equipment. These workers in a situation of poverty have no choice but take these risks. Government and other regulatory authorities need to enforce the mining industry to provide a safer working environment and assure that miners use safety equipment.

Majorities sustained multiple injuries (33.3%), and were more likely to die followed by those who sustained head/neck injuries and fractures. This nature of injuries needs immediate pre-hospital emergency care and a good hospital service, in terms of proper equipments and trained medical personnel's which is lacking in Mererani.

This study was not without limitations. Firstly, it is not considering those who didn’t report to the health centre, so the number of injuries and fatalities might be underreported. Secondly data collectors may not have collected all data, and so some data could be missing.

## Conclusion

The burden of injuries and fatalities demonstrated in this study point to the need for enhancing safety standard procedures in the mines. It is necessary to promote behavioral approaches to safety management, and particularly to motivate workers to take workplace safety seriously. It is surprising that the workers do not identify their situation as dangerous and clearly sensitization is needed. In addition to sensitization enforcement of safety regulations by government and other external regulatory agencies is needed for workers, employers and the mine owners. Mine managers should address the need to examine mining pits properly and regularly to establish their strength and durability as part of safety enforcement procedures. Penalties should be imposed for failure to provide a safe working environment and prosecutions would be appropriate when miners die due to industrial negligence. The nature of injuries reported call for improved hospital services and establishing pre-hospital emergency medical care in the country mining industry.
